# P06-01 Development of a French paper-and-pencil association test to measure athletes’ implicit doping attitudes

**DOI:** 10.1093/eurpub/ckac095.086

**Published:** 2022-08-29

**Authors:** Valentine Filleul, Fabienne d'Arripe-Longueville, Eric Meinadier, Jacky Maillot, Derwin King-Chung Chan, Stéphanie Scoffier-Mériaux, Karine Corrion

**Affiliations:** 1 Université Côte d'Azur, LAMHESS, France; 2 Medical, French Federation of Cycling, Saint-Quentin-en-Yvelines, France; 3 The Education University of Hong-Kong, Hong Kong, China

**Keywords:** implicit association test, awareness to doping, doping, sport, cycling

## Abstract

**Background:**

The continued prevalence of positive samples to banned performance-enhancing drugs confirms the importance to maintain the anti-doping efforts. Though the role of socio-cognitive variables in doping attitudes is well identified (e.g., Ntoumanis et al., 2014), the role of implicit processes remains sparsely studied in sports’ doping, especially in high level cyclists. While the potential of traditional computer-IAT has been developed to capture individuals’ implicit attitudes toward doping (Brand et al., 2014ab; Schindler et al., 2015), paper-and-pen IAT offers unquestionably ease-of-administration prospects (Chan and al., 2017). The aim of this study was thus to test and provide a preliminary validation of a French paper-and-pen IAT as an alternative method to measure implicit attitudes toward doping: the IAT-Dop.

**Method:**

This work was based on the testing procedure of the paper-and-pen Personalized Single-Category IAT test (i.e., p&p SC-IAT-P) of Bardin et al. (2016), which we enriched with the procedure of Boateng et al. (2018). This paper was built around four studies: (a) the first study consisted in the development of a preliminary version of the IAT-Dop, (b) the second study measured the dimensionality and criterion validity of the IAT-Dop to confirm the structure of the p&p version, (c) the third study verified its test-retest reliability, and (d) the fourth study explored the relations between the p&p and computerized versions of the IAT-Dop as a first approach to construct validity.

**Results:**

Study 1 developed a preliminary French version of the IAT-Dop based on Chan et al.’s IAT proposal (2017). Study 2 provided preliminary support to the dimensionality and criterion validity of the tool. Study 3 demonstrated the test-retest reliability of the instrument. Finally, Study 4 suggested the construct validity of the IAT-Dop through a significant correlation between the computerized and p&p versions.

**Conclusion:**

The IAT-Dop is a preliminary tested French-language version of a tool for measuring athletes’ implicit attitudes toward doping, with the advantages of simplicity, low cost, and quick administration. This tool should contribute to the better assessment and understanding of the mechanisms related to doping and may be a useful new indicator in the evaluation of prevention programs.

